# Nitrogen Source Screening on Agar Medium Followed by Submerged Cultivation of *Pleurotus albidus* Mycelium for Nutritional and Amino Acid Characterization

**DOI:** 10.1007/s00284-026-05119-2

**Published:** 2026-08-26

**Authors:** Eduarda Monteiro Martins Leitão, Acácio A. F. Zielinski, Patrícia Poletto

**Affiliations:** Department of Chemical and Food Engineering (EQA), Federal University of Santa Catarina (UFSC), Campus Trindade, PO box 476, Florianópolis, 88010-970 SC Brazil

## Abstract

The search for sustainable protein sources has increased interest in filamentous fungi, however, most studies focus on a limited number of established species. *Pleurotus albidus*, a basidiomycete native to South America, remains little explored for food applications, particularly regarding the nutritional characterization of its mycelium. In this study, *P. albidus* was initially cultivated on PDA agar supplemented with yeast extract (YE) or ammonium sulfate (AS) at 0.5–2 gN/L for nitrogen source screening. On agar medium, YE reduced the lag phase and accelerated plate colonization, reaching full plate coverage in 8–9 days compared to 11–12 days for AS. Based on these results, the selected condition was subsequently applied to submerged cultivation in which the mycelium grew as spherical pellets and reached 12.4 g/L after 12 days, with 23.8% protein, 26% fiber, and 39% carbohydrates. Chemical score analysis indicated that most essential amino acids (EAAs) exceeded recommended levels; however, sulfur-containing amino acids (methionine + cystine) were limiting (0.6). Threonine showed the highest score (2.3), while lysine (1.2) and tryptophan (1.1) also scored above 1.0. Non-essential amino acids such as glutamic (145 mg/g) and aspartic acid (102 mg/g) predominated. These results highlight the nutritional potential of *P. albidus* mycelium as a novel protein source and support its further investigation for future food applications, while additional studies on protein digestibility and cultivation optimization are needed to further improve amino acid balance and protein quality.

## Introduction

Over the past four decades, global demand for dietary protein has increased significantly, driven by population growth and rising income levels in emerging economies [1]. This trend has intensified concerns regarding environmental sustainability and the efficient use of natural resources, such as water, energy, and land. In response, alternative protein sources have been explored, among which fungal mycelia have received increasing attention as sustainable alternative protein sources and as promising platforms for the production of mycoproteins and other value-added food ingredients [2, 3].

Edible fungal mycelia, particularly from species of the genus *Pleurotus*, are nutritionally valuable, characterized by high protein content, low fat levels, absence of cholesterol, and the presence of essential vitamins, minerals, and bioactive compounds such as polysaccharides, triterpenoids, γ-aminobutyric acid, and ergosterol [3, 4]. In addition to their nutritional properties, these mycelia provide sensory benefits due to amino acids associated with umami flavor [5], which is relevant for the formulation of meat analogues. Another advantage lies in the feasibility of vertical mycelium cultivation, which allows for more efficient space utilization and may contribute to reduced deforestation and a lower carbon footprint associated with protein production [6].

According to the most recent Straits Research report (2026), the global mycelium market is projected to grow from USD 3.96 billion in 2026 to USD 7.60 billion by 2034, corresponding to a compound annual growth rate (CAGR) of 8.5%. This growth is driven by the multiple applications of mycelium: besides the food sector, it is used in industries such as beverages, packaging, clothing, and animal feed, consolidating its position as a versatile resource. Within the food sector, mycelium also stands out as a sustainable alternative to meat, particularly with the rise of veganism.

Among established examples of mycelium use in food, *Fusarium venenatum*, employed by the company Quorn^®^, contains approximately 45% protein, 25% fiber, and low fat content, in addition to a digestibility (PDCAAS) close to 1.0 [7]. Similarly, *Neurospora crassa*, used by Meati Foods^®^, presents around 45% protein and 30% fiber, with comparable digestibility [1]. Despite these promising features, the production and consumption of mycelium-based proteins still face challenges, particularly regarding consumer acceptance and the limited availability of technical and scientific data on their nutritional composition and functional properties [8, 9].

In this context, *P. albidus* mycelium emerges as a promising yet underexplored candidate, showing favorable growth under submerged culture conditions [10]. Therefore, the present study aimed to investigate the growth behavior of *P. albidus* under different nitrogen sources and concentrations in solid medium as a preliminary screening step, in order to select suitable conditions to be subsequently applied to submerged cultivation. The resulting biomass was then evaluated in terms of yield, proximate composition, and amino acid profile, focusing on its applicability as a sustainable and alternative protein source for food applications.

## Materials and Methods

### Fungal Strain and Maintenance

*P. albidus* strain (88 F.13), originally isolated from the Pampa and Atlantic Forest biomes (São Francisco de Paula, RS, Brazil), was provided by the Enzymes and Biomass Laboratory of the Biotechnology Institute at the University of Caxias do Sul. The strain is registered in the university’s Herbarium [11]. The culture was maintained on potato dextrose agar (PDA; Kasvi, Brazil) by biweekly subculturing using 5 mm mycelial plugs. Plates were incubated at 28 °C in the absence of light and subsequently stored at 4 °C. Culture integrity was periodically monitored to detect possible contaminations.

### Evaluation of Mycelium Cultivated on Agar Surface

#### Effect of Nitrogen Source and Concentration on Radial Growth

To investigate the effect of nitrogen source and concentration on *P. albidus* growth, PDA medium was supplemented with either ammonium sulfate (AS; NH₄)₂SO₄, analytical grade, Êxodo Científica, Brazil) or yeast extract (YE; Kasvi, Brazil). According to the manufacturer’s specifications, the product consists of the water-soluble fraction of autolyzed *Saccharomyces cerevisiae* cells and is rich in amino acids, B-complex vitamins, and other growth factors. The product contains a minimum total nitrogen content of 10% and amino nitrogen ranging from 4.5 to 5.4%.

The supplementation levels were standardized to provide nominally equivalent amounts of total nitrogen (0.5, 1.0, 1.5, and 2.0 g_N_/L), enabling a comparative evaluation of the effects of organic and inorganic nitrogen enrichment on fungal growth. Nitrogen concentrations were calculated based on the molecular composition for AS and on the total nitrogen content reported by the manufacturer for YE.

After sterilization (121 °C for 15 min), a volume of 30 mL of medium was manually dispensed into each 90 mm Petri dish to ensure volume consistency for subsequent biomass yield calculations (mg/plate). Each plate was then inoculated with two 5 mm mycelial discs, previously cultivated on standard PDA medium (7 days of incubation at 28 °C), centrally positioned. The plates were incubated at 28 °C for 15 days in the dark in a BOD incubator (SL-200/364, Solab, Brazil). The experiment was conducted in quadruplicate. Radial growth, at all concentrations (0.5, 1.0, 1.5, and 2.0 g_N_/L) of YE and AS, was monitored daily through manual measurements and image analysis using ImageJ software. Unsupplemented PDA was used as the control.

The maximum specific radial growth rate (µ_Xmax_) was determined for each replicate from the slope of the linear regression of the natural logarithm of radial growth versus incubation time during the exponential growth phase. The exponential phase was identified as the interval showing an approximately linear relationship after natural logarithm transformation of the radial growth data. The resulting µ_Xmax_ values were expressed in day⁻¹ and used for statistical comparison among cultivation conditions.

#### Dry Biomass Yield

After 15 days of incubation, fungal biomass was carefully removed from the plate surface and washed three times with distilled water heated to 50 °C to remove residual culture medium. The material was then frozen at −80 °C and subsequently freeze-dried using a lyophilizer (L101, Liotop, Brazil). Dry biomass was quantified and expressed as mg per plate for each evaluated condition.

#### Total Nitrogen and Protein Content

Total nitrogen content was determined using a Kjeldahl digestion and distillation system (SL-81/40, Solab, Brazil) following the AOAC official protocol (method nº 954.01), using the freeze-dried mycelial biomass obtained as described in Sect. Dry Biomass Yield. The crude protein content was estimated using a nitrogen-to-protein conversion factor of 4.38 [12].

### *P. albidus* Production in Submerged Culture

Cultivation on solid media was used to determine the most suitable nitrogen source and concentration, based on biomass yield, growth rate, and protein content. Submerged culture was then carried out under the optimized condition to obtain fungal biomass.

The culture medium consisted of glucose (20 g/L), yeast extract supplying 1.5 g_N_/L, and 5% (v/v) of a modified mineral solution [13], composed of: KH₂PO₄ (2.0 g), (NH₄)₂SO₄ (1.3 g), urea (0.3 g), MgSO₄·7 H₂O (0.3 g), CaCl₂ (0.3 g), FeSO₄·7 H₂O (0.05 g), MnSO₄·H₂O (0.0156 g), ZnSO₄·7 H₂O (0.014 g), and CoCl₂·6 H₂O (0.02 g) per liter of distilled water. The initial pH of the culture medium was adjusted to 7.0. Based on the composition of the medium, the total theoretical nitrogen concentration was approximately 1.52 g/L, including minor contributions from ammonium sulfate and urea in the mineral solution.

In 500 mL Erlenmeyer flasks, 250 mL of medium was added and sterilized at 121 °C for 15 min. After cooling, the medium was inoculated with three 5 mm mycelial discs. Flasks were incubated at 28 °C in an orbital shaker (TE-424, Tecnal, Brazil) at 180 rpm. Triplicates were prepared, and each replicate was fully harvested at 0, 3, 6, 9, 12, and 15 days of fermentation for glucose consumption and biomass yield analysis.

#### Glucose Consumption Quantification

Glucose concentration in the culture medium was determined using the 3,5-dinitrosalicylic acid (DNS) method, as described by Miller [14], with modifications. A standard calibration curve was constructed using glucose concentrations ranging from 0.1 to 4.0 g/L. Absorbance was measured at 540 nm using a microplate reader (Epoch™, BioTek Instruments, USA). The calibration curve was linear within this range, following the equation *y = 0.1894x − 0.0198* (R² = 0.9965), where *y* is the absorbance and *x* is the glucose concentration (g/L).

#### Biomass Yield Determination

Biomass yield was determined by a gravimetric method. The culture broth was transferred to 50 mL Falcon tubes (previously identified and tared) and centrifuged at 3,000 × g for 20 min using a centrifuge (SL-707, Solab, Brazil). The supernatant was reserved for further analyses, while the cell pellet was resuspended in 25 mL of distilled water and subjected to a second centrifugation under the same conditions. After the second step, the supernatant was discarded, and the tube containing the cell pellet was transferred to an oven at 105 °C for 24 h, followed by cooling in a desiccator to constant weight, according to AOAC method nº 925.10. The dry weight obtained was used to calculate biomass yield.

#### Fungal Pellet Diameter Measurement

Fungal pellets recovered from the submerged culture according to the procedure described in Sect. Biomass Yield Determination were photographed after cultivation, and pellet diameter distribution was determined by image analysis using ImageJ software. A total of 64 pellets were measured.

#### Kinetic Parameters, Yield Coefficients, and Biomass Productivity

The maximum specific growth rate (µ_Xmax_) was determined from the slope of the linear regression of the natural logarithm of biomass concentration versus cultivation time during the exponential growth phase.

The substrate-to-biomass conversion yield (Y_X/S_) was calculated as the mass of mycelial biomass produced per mass of substrate consumed (g/g), according to Eq. 1. Biomass productivity (Pₓ) was expressed as the amount of biomass produced per unit of time (g/L/day), as shown in Eq. 2.

1 $$\:{Y}_{X/S}=\:\frac{{X}_{f}-{X}_{i}}{{S}_{i}-{S}_{f}}$$

2 $$\:{P}_{X}=\:\frac{Xf-Xi}{tf-ti}$$

where X_f_ and X_i_ are the final and initial biomass concentrations (g/L); S_i_ and S_f_ are the initial and final glucose concentrations (g/L); t_f_ and t_i_ are the final and initial cultivation times (day), respectively.

The apparent nitrogen-to-biomass yield (Y_X/N_) was calculated as the mass of mycelial biomass produced per mass of nitrogen consumed (g/g), according to Eq. 3. The apparent nitrogen-to-protein yield (Y_Prot/N_) was estimated as the mass of protein produced per mass of nitrogen consumed (g/g), according to Eq. 4.

3 $$\:{Y}_{X/N}=\frac{{X}_{f}}{{N}_{i}-{N}_{f}}$$

4 $$\:{Y}_{Prot/N}=\frac{Prot}{{N}_{i}-{N}_{f}}$$

where N_i_ and N_f_ are the initial and final nitrogen concentrations (g_N_/L), respectively, and Prot is the final protein concentration in the biomass (g/L), estimated from biomass concentration and protein content.

### Nutritional Characterization of Mycelial Biomass from Submerged Cultivation

#### Biomass Recovery and Preparation

To determine the nutritional profile and amino acid composition, a dedicated submerged cultivation was performed. The culture broth was thermally treated at 50 °C for 1 h to inactivate the mycelium, following a modified methodology adapted from [15]. Inactivation was confirmed by the absence of growth after inoculating treated biomass aliquots onto PDA plates. The biomass was then rinsed with distilled water, recovered by filtration, manually pressed to remove excess liquid, and stored at −80 °C before being freeze-dried for subsequent analyses.

#### Proximate Composition

The proximate composition of the mycelial biomass, including lipids (AOAC 954.02), crude protein (AOAC 954.01), fiber (AOAC 978.10), ash (AOAC 923.03), and carbohydrates (calculated by difference), was determined according to the official AOAC methods. All results were expressed on a dry weight basis.

The caloric value was calculated using the Atwater conversion factors: 4 kcal·g⁻¹ for proteins, 4 kcal·g⁻¹ for carbohydrates, and 9 kcal·g⁻¹ for lipids. All analyses were performed in triplicate.

#### Amino Acid Profile

The amino acid profile analysis of the biomass was carried out at CBO Análises Laboratoriais (Valinhos, SP, Brazil). The amino acid profile of the mycelium was determined by high-performance liquid chromatography (HPLC) with pre-column derivatization using phenylisothiocyanate (PITC), following the Pico-Tag system protocol (Waters) [16]. Samples were subjected to acid hydrolysis with 6 N HCl containing 0.1% phenol, in the presence of norleucine as an internal standard, for 24 h at 110 °C. After filtration, the hydrolysates were purified using Sep-Pak C18 cartridges, evaporated under vacuum, redissolved, and derivatized with PITC. The phenylthiocarbamyl (PTC) derivatives were separated on a C18 column (Nova-Pak, 3.9 × 150 mm) using an acetonitrile/acetate buffer gradient (pH 6.4), with absorbance detection at 254 nm.

Tryptophan content was determined after alkaline hydrolysis following the methodology described by Lucas & Sotelo [17]. Approximately 100 mg of dry sample were hydrolyzed with 4 N LiOH in culture tubes with Teflon caps, under a nitrogen atmosphere. Hydrolysis was carried out at 145 °C for 6 h. After cooling, the samples were neutralized with phosphoric acid (85%), diluted in phosphate buffer (0.2 M, pH 7.0), and filtered.

To assess the nutritional quality of the mycelium protein, the essential amino acid (EAA) score was calculated according to Eq. 5:

5 $$\:EAA=\frac{\mathrm{m}\mathrm{g}\:\mathrm{o}\mathrm{f}\:\mathrm{a}\mathrm{m}\mathrm{i}\mathrm{n}\mathrm{o}\:\mathrm{a}\mathrm{c}\mathrm{i}\mathrm{d}\mathrm{s}\:\mathrm{p}\mathrm{e}\mathrm{r}\:1\:\mathrm{g}\:\mathrm{o}\mathrm{f}\:\mathrm{p}\mathrm{r}\mathrm{o}\mathrm{t}\mathrm{e}\mathrm{i}\mathrm{n}}{\mathrm{m}\mathrm{g}\:\mathrm{o}\mathrm{f}\:\mathrm{a}\mathrm{m}\mathrm{i}\mathrm{n}\mathrm{o}\:\mathrm{a}\mathrm{c}\mathrm{i}\mathrm{d}\:\mathrm{p}\mathrm{e}\mathrm{r}\:1\:\mathrm{g}\:\mathrm{o}\mathrm{f}\:\mathrm{r}\mathrm{e}\mathrm{f}\mathrm{e}\mathrm{r}\mathrm{e}\mathrm{n}\mathrm{c}\mathrm{e}\:\mathrm{p}\mathrm{r}\mathrm{o}\mathrm{t}\mathrm{e}\mathrm{i}\mathrm{n}}$$

The reference protein used was the one established by FAO/WHO for high biological value proteins [18].

### Statistical Analysis

Results were expressed as mean ± standard deviation. Agar plate experiments were performed in biological quadruplicate. Submerged cultivation experiments were carried out in biological triplicate using three independent flasks for each sampling point. For proximate composition and amino acid analyses, biomass obtained from multiple submerged culture flasks was pooled to provide sufficient material. Proximate composition determinations were performed in analytical triplicate, whereas the amino acid profile was determined in a single analytical measurement. Data were analyzed by one-way analysis of variance (ANOVA), followed by Tukey’s honestly significant difference (HSD) test for multiple comparisons at a significance level of 5% (*p* < 0.05). Prior to ANOVA, the assumptions of normality of residuals and homogeneity of variances were assessed using normal probability plots of residuals and Levene’s test, respectively. All statistical analyses were performed using Statistica v. 13.5 (TIBCO Software Inc., Palo Alto, CA, USA).

## Results and Discussion

### Growth Curve Monitoring of *P. albidus* Under Different Nitrogen Sources and Concentrations

PDA medium cultivation was employed as a preliminary screening approach to evaluate the effect of different organic and inorganic nitrogen sources supplied at equivalent nitrogen concentrations on the growth kinetics of *P. albidus*, aiming at selecting the most favorable condition for subsequent application in submerged cultivation. Since potato infusion inherently contains some organic nitrogen and micronutrients, this setup serves as a comparative evaluation of nitrogen supplementation effects rather than an absolute nitrogen utilization assay.

**Fig. 1:**
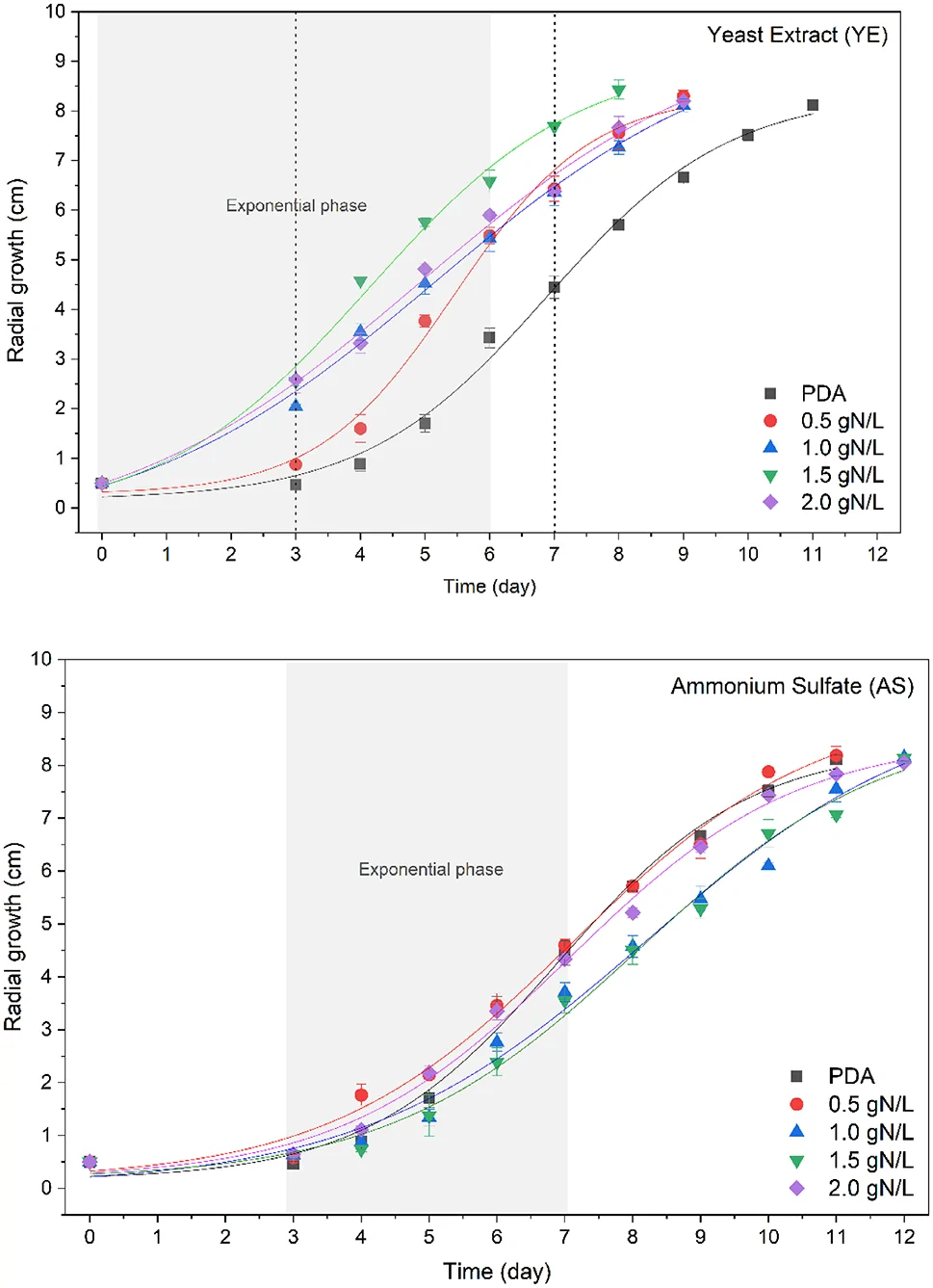
Radial growth of *P. albidus* cultivated on PDA supplemented with different nitrogen concentrations provided as yeast extract (YE) or ammonium sulfate (AS). Symbols represent mean values ± standard deviation (*n* = 4), and curves correspond to Boltzmann sigmoidal fits (R^2^ > 0.98). Shaded regions indicate the experimental intervals identified as the exponential growth phase (see Sect. Effect of Nitrogen Source and Concentration on Radial Growth). For YE-supplemented media (1.0–2.0 g_N_/L), the exponential phase was considered between 0–6 days, whereas for YE 0.5 gN/L, PDA (dotted vertical lines) and all AS-supplemented media, it was considered between days 3 and 7

Figure 1 shows the radial growth of *P. albidus* mycelium on PDA medium supplemented with yeast extract (YE) and ammonium sulfate (AS), at nitrogen-equivalent concentrations, compared to the control sample (unsupplemented PDA). Although equivalent total nitrogen concentrations were employed, YE also provides amino acids, peptides, vitamins and other growth-promoting compounds [19, 20] that are absent from AS. The initial inoculum diameter was recorded as the time-zero measurement, and radial growth was subsequently monitored every 24 h from the third day onward. Boltzmann sigmoidal fitting was applied only as a descriptive tool to facilitate visualization of the radial growth behavior, while µ_Xmax_ was calculated directly from the experimental data obtained during the exponential growth phase.

The radial growth curves of *P. albidus* indicate distinct responses to the nitrogen sources tested. The growth curves show that *P. albidus* cultured on media supplemented with YE exhibited faster growth, reaching the exponential (log) phase as early as the third day of cultivation, compared to the control sample (PDA only). Mycelium grown with YE supplementation covered the plate surface more rapidly than that grown with AS, achieving complete colonization after 8–9 days. In contrast, all AS-supplemented media required 12 days to reach the edge of the plate, similarly to the unsupplemented control.

The superior performance observed for YE can be attributed to its complex composition, which provides readily assimilable nutrients that facilitate fungal growth [19, 20]. Therefore, the observed differences should not be interpreted exclusively as effects of nitrogen equivalence on a g_N_/L basis. Organic nitrogen compounds can be directly absorbed through amino acid permeases and peptide transport systems, facilitating rapid incorporation into cellular metabolism [21, 22]. In contrast, AS provides nitrogen exclusively in inorganic form (NH₄⁺), requiring metabolic conversion into glutamate and glutamine through ammonia assimilation pathways involving enzymes such as GDH, GS, and GOGAT [23]. This additional metabolic demand may increase the energetic cost of nitrogen assimilation and contribute to the slower colonization observed in AS-supplemented media.

Table 1 presents the µ_Xmax_, determined from the slope of the linear regression of the natural logarithm of radial growth during the exponential growth phase identified in Fig. 1.

**Table 1:** Maximum specific radial growth rate (µ_Xmax_) of *P. albidus* cultivated on PDA medium supplemented with yeast extract (YE) or ammonium sulfate (AS) at nitrogen-equivalent concentrations

Nitrogen concentration (g_*N*_/L)	YE		AS	
	µ_Xmax_ (day^−1^)	^‡^Time to full plate colonization (days)	*µ_Xmax_ (day^−1^)	^‡^Time to full plate colonization (days)
0.5	*0.63 ± 0.09^aA^	9	0.48 ± 0.02^bB^	11
1	0.47 ± 0.02^bB^	9	0.47 ± 0.03^bB^	11
1,5	0.51 ± 0.004^bA^	8	0.47 ± 0.02^bA^	12
2	0.47 ± 0.02^bA^	9	0.49 ± 0.02^bA^	12
Control (PDA)	*0.61 ± 0.06^aA^	11	0.61 ± 0.06^aA^	11

*Calculated from the exponential growth phase after day 3. ^‡^Plate colonization was defined as complete coverage of the agar surface by the mycelium. Values are presented as mean ± standard deviation (*n* = 4). Different lowercase letters in the same column indicate statistically significant differences between nitrogen concentrations (Tukey test, *p* ≤ 0.05). Different uppercase letters in the same row indicate statistically significant differences between nitrogen sources at the same concentration (Tukey test, *p* ≤ 0.05)

While Table 1 shows that the control (PDA) and YE at 0.5 g_N_/L yielded the highest values of µ_Xmax_ (0.61 and 0.63 day⁻¹, respectively), these values alone do not fully reflect the actual performance of the mycelium under the tested conditions. Both conditions exhibited a detectable lag phase before the onset of exponential growth. In contrast, YE at 1.5 g_N_/L showed a lower µ_Xmax_ (0.51 day⁻¹) but displayed no lag phase and achieved complete plate colonization one day earlier than YE at 0.5 g_N_/L.

These results demonstrate that µ_Xmax_, calculated solely from the exponential phase, may not represent the overall growth dynamics when lag phase duration is not considered. Although YE 0.5 g_N_/L presented the highest calculated µ_Xmax_, the delayed growth initiation reduced its overall efficiency in terms of plate colonization time. On the other hand, YE at 1.5 g_N_/L allowed for immediate and sustained growth, leading to faster colonization despite its lower µ_Xmax_. YE-supplemented media at concentrations between 1.0 and 2.0 g_N_/L did not exhibit significant differences in µ_Xmax_. Apparently, YE reduced or eliminated the adaptation phase, promoting faster colonization, consistent with the metabolic advantages previously discussed for complex organic nitrogen sources [20]. As growth begins actively from the start, the log phase may be shorter or less steep.

In contrast, the AS-containing medium demonstrated poorer overall mycelial growth performance, since all tested concentrations exhibited a lag phase and a growth pattern closely resembling that of the control. These results indicate that AS assimilation by the mycelium is less efficient compared to organic sources like YE. Furthermore, ammonium ion assimilation can promote medium acidification by releasing H⁺ ions, which lowers the pH and may influence mycelial growth and metabolism [24, 25]. High AS concentrations may negatively affect fungal growth through combined osmotic and metabolic effects. Increased ionic strength can reduce water availability, impair nutrient transport, and increase the energetic demand required to maintain cellular homeostasis [26]. These combined factors explain the higher time for plate colonization observed in AS treatments, despite their µ_Xmax_ being close to that of YE. Therefore, the present comparison should be interpreted as an evaluation of organic (YE) and inorganic (AS) nitrogen supplementation strategies supplied at equivalent nitrogen concentrations, rather than as an assessment of nitrogen availability alone.

**Fig. 2:**
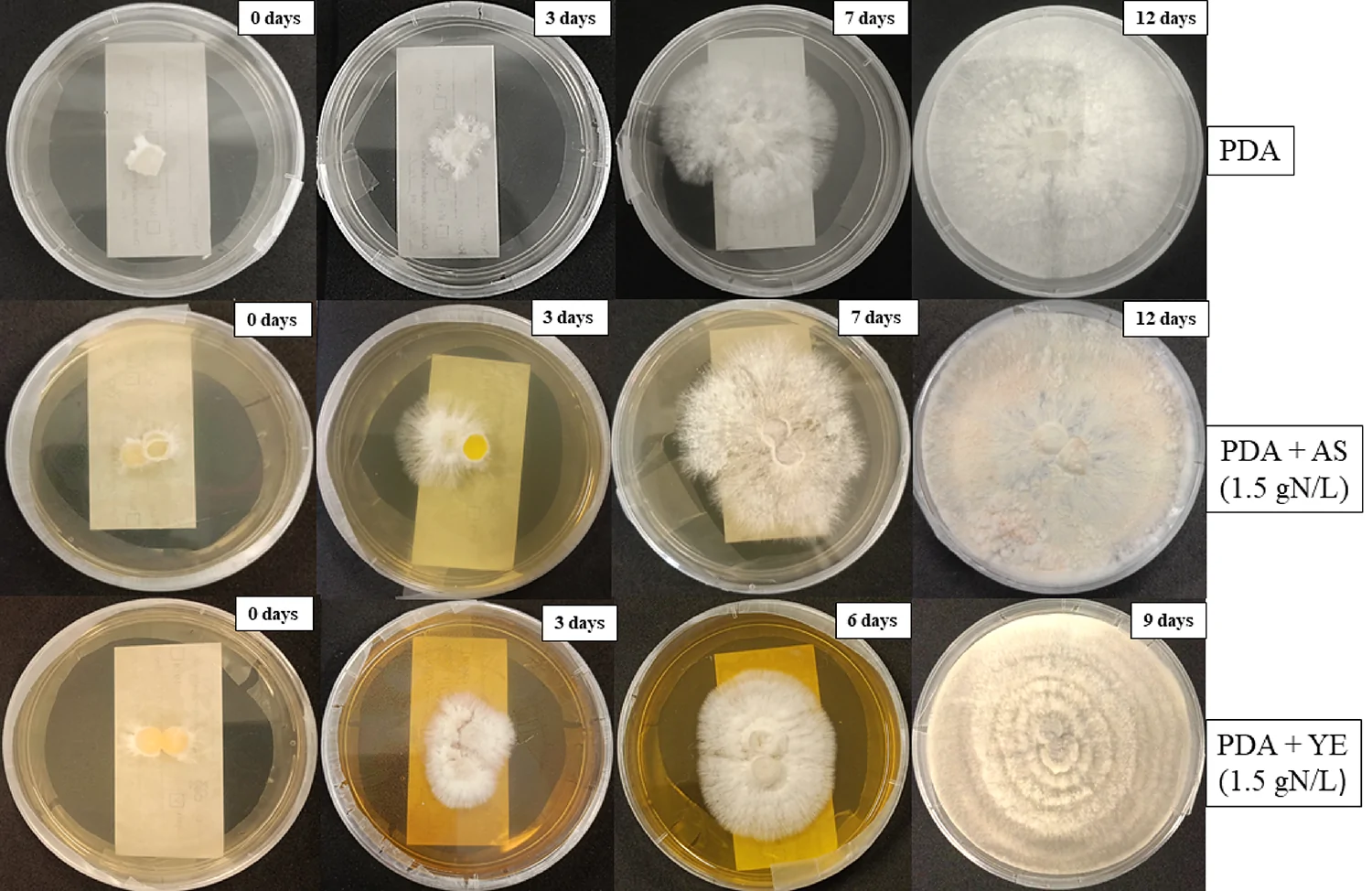
Photographs of *P. albidus* mycelial growth over the cultivation period (0 to 12 days) in media supplemented with yeast extract (YE) and ammonium sulfate (AS) (1.5 g_N_/L of nitrogen), compared to the control medium (PDA). Images correspond to representative quadruplicate cultivations. Petri dishes had an internal diameter of approximately 8 cm

Qualitative monitoring of mycelial growth through photographic records (Fig. 2) also revealed morphological differences associated with the type of nitrogen supplement. Mycelium cultivated with YE (1.5 g_N_/L) showed denser growth, exhibiting a distinct zonal growth pattern, characterized by concentric rings of mycelial density, whereas mycelium grown with AS at the same nitrogen concentration (1.5 g_N_/L) exhibited a sparser structure with thinner hyphal filaments and a less compact distribution. This observation suggests that nitrogen source complexity also influences the spatial organization of mycelial development.

### Protein Content and Biomass Yield as a Function of Nitrogen Source and Concentration

Table 2 presents the biomass yield (mg/plate) and protein content (% w/w) of *P. albidus* mycelium cultivated on PDA (control) and on media supplemented with yeast extract (YE) or ammonium sulfate (AS), at equivalent total nitrogen concentrations (0.5 to 2.0 g_N_/L). The data indicate that both biomass yield and protein content were significantly influenced by the nitrogen source and concentration (*p* ≤ 0.05).

**Table 2:** Protein content and biomass yield of *P. albidus* mycelium cultivated on PDA (control) and on media supplemented with yeast extract (YE) or ammonium sulfate (AS), at equivalent nitrogen concentrations. Data refer to the mycelial biomass collected from the agar surface and expressed on a dry weight basis

Nitrogen concentration (g_*N*_/L)	YE		AS	
	Protein (%)	Yield (mg/plate)	Protein (%)	Yield (mg/plate)
0.5	21.3 ± 0.3^cA^	148 ± 14^cB^	15.8 ± 0.8^cB^	205 ± 13^aA^
1	25.8 ± 0.1^bA^	260 ± 7^bA^	20.8 ± 0.2^bB^	172 ± 3^bB^
1.5	30.1 ± 0.4^aA^	270 ± 2^bA^	27.9 ± 0.6^aA^	181 ± 7^bB^
2	32.1 ± 0.3^aA^	308 ± 5^aA^	30.8 ± 1^aA^	211 ± 7^aB^
Control (PDA)	15 ± 0.7^dA^	157 ± 3^cB^	15 ± 0.7^cA^	157 ± 3^cB^

Values are presented as mean ± standard deviation (*n* = 4). Different lowercase letters in the same column indicate significant differences between nitrogen concentrations within the same medium (Tukey’s test, *p* ≤ 0.05). Different uppercase letters in the same row indicate significant differences between nitrogen sources at the same concentration (Tukey’s test, *p* ≤ 0.05).

In the control medium (PDA), without additional nitrogen supplementation, biomass yield and protein content were 157 mg/plate and 15%, respectively. Nitrogen supplementation significantly increased both parameters. Protein content progressively increased with the nitrogen concentration supplied by YE, reaching 32.1% at 2 g_N_/L. A similar result was obtained with AS, where the protein content was 30.8% at the same concentration. For both nitrogen sources, protein contents at 1.5 and 2.0 gN/L did not differ significantly, suggesting that protein enrichment approached a plateau above 1.5 gN/L.

The yield of *P. albidus* showed distinct responses depending on the nitrogen source tested. In the YE medium, the yield increased proportionally with the nitrogen concentration, reaching 308 mg/plate at 2 g_N_/L, a value significantly higher than the others (*p* ≤ 0.05). In the AS medium, the maximum yield was also observed at 2 g_N_/L (211 mg/plate), but it was lower than that obtained with YE, suggesting that although both sources promote growth, YE provides more favorable conditions for biomass accumulation.

The present results are consistent with previous findings showing that yeast extract in submerged cultivation of *P. sajor-caju* enhanced biomass production, whereas AS, at high concentrations, inhibited mycelial growth but was more effective at moderate levels [27]. The higher biomass yields obtained with YE are consistent with its complex nutritional composition, as discussed in Sect. Growth Curve Monitoring of *P. albidus *Under Different Nitrogen Sources and Concentrations. This nutritional complexity likely contributes to the greater biomass accumulation observed relative to AS [28]. Previous studies on *Pleurotus* species have also highlighted the benefits of yeast extract in biomass production [29, 30], reinforcing the applicability of this supplement in optimized formulations for mycelial cultivation.

### Growth of *P. albidus* Mycelium in Submerged Culture

**Fig. 3:**
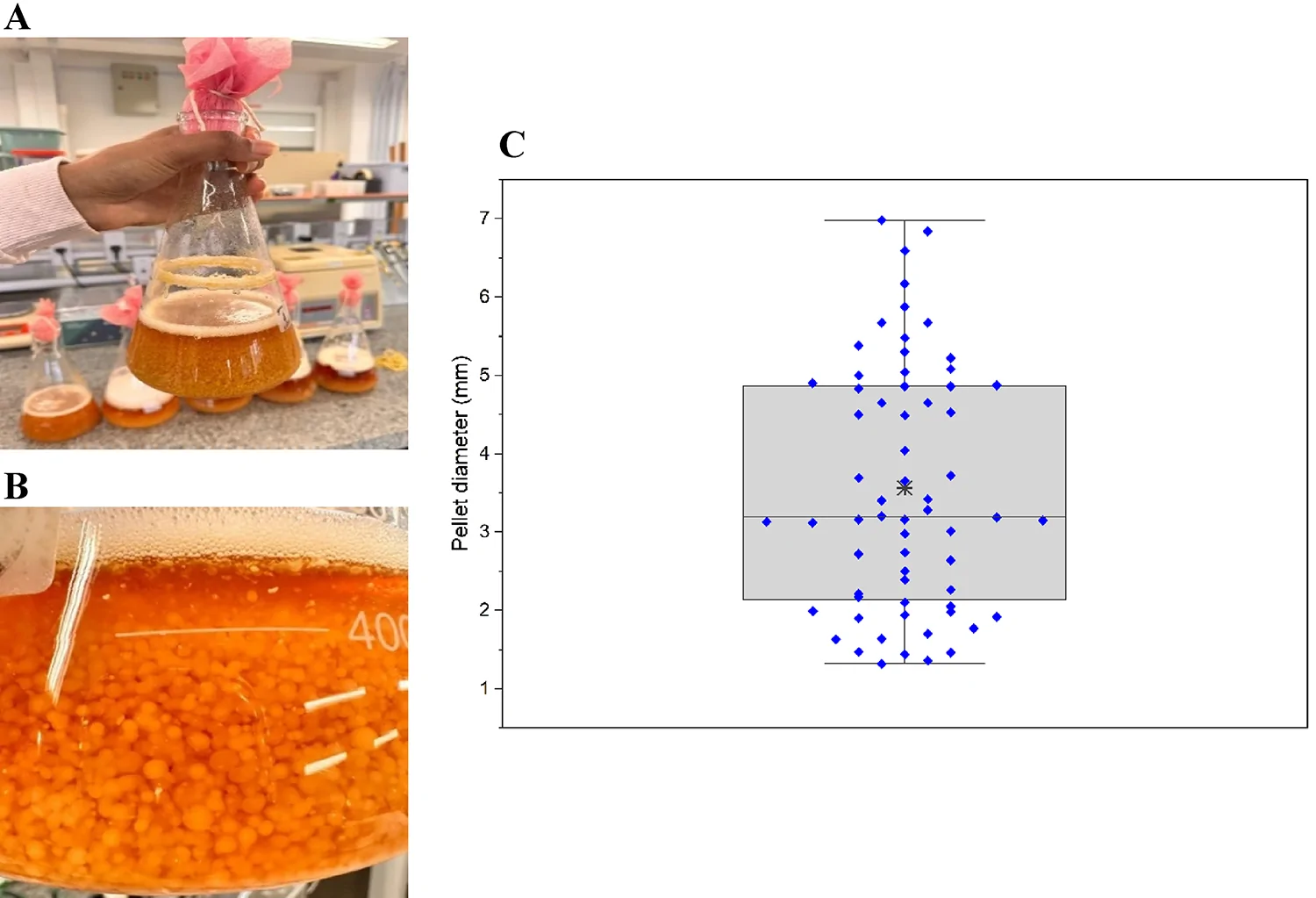
Submerged cultivation of *P. albidus* after 15 days: (A) Erlenmeyer flask containing the cultivation medium with visible mycelial pellets; (B) close-up view of the flask, highlighting the high density of suspended pellets; (C) distribution of pellet diameters measured by ImageJ image analysis (*n* = 64 pellets). Pellet diameters ranged from 1.32 to 6.92 mm. The box represents the interquartile range, the central line indicates the median, whiskers indicate minimum and maximum values, dots represent individual measurements, and the cross symbol represents the mean diameter (3.56 mm)

The submerged cultivation of *P. albidus* was carried out over 15 days using a nitrogen concentration of 1.5 g_N_/L provided by yeast extract, based on the results obtained in the previous section. This condition was selected because it exhibited the shortest plate colonization time, while achieving a protein content that was not significantly different from that obtained with 2.0 g_N_/L. Although 2.0 g_N_/L resulted in the highest biomass yield, 1.5 g_N_/L was considered the most suitable condition, as it provided comparable protein enrichment with a lower yeast extract concentration. After cultivation, intense biomass formation was observed, with the development of suspended mycelial pellets distributed throughout the culture medium (Fig. 3A–B). The culture broth exhibited a high density of pellets with relatively heterogeneous morphology and size distribution.

Pellets exhibited a whitish-yellow coloration and diameters ranging from 1.32 to 6.92 mm. Based on ImageJ analysis of 64 pellets (Fig. 3C), the average pellet diameter was 3.56 ± 1.51 mm, indicating considerable size variability during submerged cultivation. These pellets are characterized by spherical mycelial aggregates composed of branched and partially entangled hyphal networks [8, 31]. Pellet formation, as observed in this study, facilitates biomass recovery, reduces the viscosity of the medium, and may improve process control.

Similar observations have been reported for *Pleurotus ostreatus*, in which pellet diameter was strongly influenced by agitation conditions and associated with differences in pellet structure and biological activity. Kim and Song [30] classified fungal pellets as small (1–2 mm), medium (~ 3 mm), and large (4–5 mm), noting that lower agitation rates (100 rpm) favored the formation of larger pellets due to reduced shear stress, whereas higher speeds (150 rpm) resulted in smaller, denser structures. These morphological differences are particularly relevant because pellet size and structure directly affect mass transfer phenomena during submerged cultivation.

Smaller pellets tend to allow better diffusion of oxygen and nutrients into the inner regions of the structure, promoting higher metabolic activity and more homogeneous growth. In contrast, larger pellets may develop internal gradients of oxygen and substrate, leading to central regions with oxygen limitation or even metabolic inactivity, which can reduce the overall efficiency of the process. Therefore, although pellet formation offers operational advantages, controlling pellet size and structure is essential to optimize mass transfer and maximize mycelial productivity [31].

### Kinetic Parameters of *P. albidus* Mycelium in Submerged Cultivation

During the 15 days of submerged cultivation, glucose consumption (as the carbon source), nitrogen concentration and mycelial biomass accumulation on a dry weight basis were monitored.

**Fig. 4:**
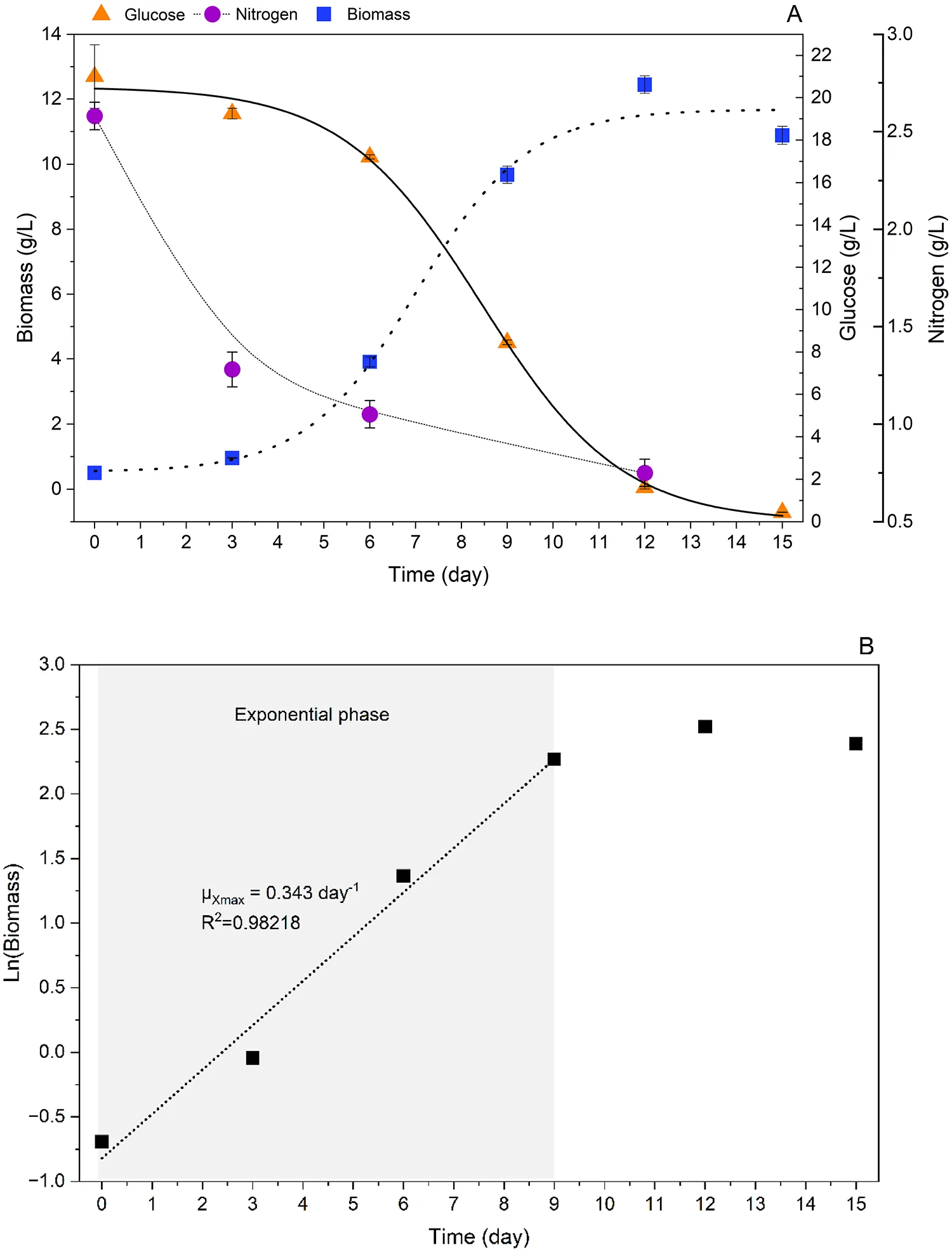
Kinetic profiles of *P. albidus* submerged fermentation over 15 days. **(A)** Mycelial biomass, glucose consumption, and nitrogen uptake. Lines for biomass and glucose represent Boltzmann sigmoidal fits (R^2^ > 0.97), while the nitrogen curve is presented as a visual guide to indicate the consumption trend. **(B)** Linear regression of the natural logarithm of biomass during the exponential phase (0–9 days) (R^2^ = 0.98), in which the slope corresponds to the maximum specific growth rate (µXmax) calculated from the shaded region. Error bars represent standard deviation of three independent cultivations (*n* = 3); where not visible, they are smaller than the symbols

The glucose consumption profile (Fig. 4A) showed an initial period of lower consumption rate during the first three days, in which glucose decreased from 21.0 to 19.8 g/L. From day 3 onward, there was an increase in the consumption rate, with glucose levels dropping to 8.9 g/L by day 9. In the following days, between day 9 and day 15, the consumption rate gradually declined until reaching just 0.5 g/L, suggesting depletion of the available carbon source and a stabilization of growth.

Biomass accumulation closely followed the substrate consumption profile (Fig. 4A). From the beginning of cultivation, a continuous increase in biomass was observed, with no detectable lag phase, as confirmed by the linear regression of the natural logarithm of biomass over time (Fig. 4B). Between days 0 and 9, biomass increased rapidly, reaching 9.7 g/L. Although glucose consumption was initially slower during the first three days, biomass accumulation started immediately, which may be associated with the utilization of intracellular reserves and nutrients carried over from the inoculum. In addition, physiological adaptation of glucose transport and assimilation systems may have contributed to the delayed increase in glucose uptake observed after day 3 [32, 33].

The maximum specific growth rate (µ_Xmax_) was determined from the slope of the linear regression applied to the exponential phase (days 0 to 9), resulting in µ_Xmax_ = 0.343 day⁻¹ (R² = 0.98). This result indicates that the inoculum was metabolically active at the start of cultivation, requiring minimal adaptation to the culture conditions.

The maximum biomass concentration (12.4 g/L) was reached on day 12, suggesting the onset of the stationary phase, likely due to the depletion of available glucose. Although residual nitrogen was still present, a slight decline to 10.9 g/L observed by day 15 may be attributed to cellular autolysis or the limitation of other essential nutrients.

Nitrogen analysis revealed a progressive decrease in concentration throughout cultivation (Fig. 4A). The initial nitrogen concentration of 2.58 g/L decreased to 0.75 g/L by day 12, coinciding with the maximum biomass concentration. This behavior indicates active nitrogen assimilation during the exponential growth phase, highlighting its role in supporting mycelial growth.

To further quantify the relationships between substrate consumption, biomass formation, and nitrogen utilization, the main kinetic and conversion parameters are summarized in Table 3.

**Table 3:** Kinetic and conversion parameters of *P. albidus* submerged cultivation at 12 days

Parameter	Value	Unit
Initial glucose (S_i_)	21.0	g/L
Final glucose (S_f_)	1.6	g/L
Glucose consumed	19.4	g/L
Final biomass (X_f_)	12.4	g/L
Maximum specific growth rate (µ_Xmax_)	0.34	day⁻¹
Biomass productivity (P_X_)	1.0	g/L/day
Apparent substrate-to-biomass yield (Y_X/S_)	0.63	g/g
*Initial nitrogen (N_i_)	2.58	g/L
Final nitrogen (N_f_)	0.75	g/L
Nitrogen consumed	1.83	g/L
Nitrogen utilization	71	%
Apparent nitrogen-to-biomass (Y_X/N_)	6.8	g/g
Protein content in X_f_	27.0	% (d.b.)
Estimated protein concentration	3.3	g/L
Apparent nitrogen-to-protein yield (Y_Prot/N_)	1.8	g/g
**Apparent N recovery as protein	42	%

*Measured by the Kjeldahl method. **Estimated from Y_Prot/N_ using the nitrogen-to-protein conversion factor (4.38). Kinetic parameters were calculated from the mean kinetic profiles of three independent cultivations (*n* = 3)

Compared with the glucose-based medium evaluated by Kirsch et al. [10], the present study showed higher final biomass formation, despite similar biomass productivity values. The authors reported a Y_X/S_ of 0.43 g/g and productivity of 1.3 g/L/day for glucose-based cultivation, whereas the present study achieved a Y_X/S_ of 0.63 g/g and productivity of 1.0 g/L/day. These differences may be associated with variations in medium composition and cultivation conditions.

In addition to carbon consumption, the present study also evaluated nitrogen assimilation during submerged cultivation. Nitrogen utilization reached approximately 71%, indicating substantial nitrogen uptake throughout the exponential growth phase. The apparent Y_X/N_ and Y_Prot/N_ further suggest the incorporation of nitrogen into biomass and protein synthesis. Based on the estimated Y_Prot/N_, approximately 42% of the consumed nitrogen was recovered as protein-associated nitrogen in the final biomass, while the remaining fraction was likely distributed among other cellular components and metabolites.

Although residual nitrogen remained in the medium at the end of cultivation, biomass accumulation decreased after day 12, suggesting that growth limitation was more strongly associated with glucose depletion and metabolic regulation than with complete nitrogen exhaustion. Therefore, the results obtained here expand the currently limited information on *P. albidus* submerged cultivation by integrating substrate consumption, nitrogen assimilation, and biomass formation into a single kinetic analysis.

### Proximate Composition of *P. albidus* Mycelial Biomass Cultivated by Submerged Fermentation

The proximate composition of *P. albidus* mycelial biomass after thermal inactivation and drying is presented in Table 4. The biomass exhibited a low lipid content (3.4%) and an ash content (7.7%), while its main constituents were carbohydrates (39.0%), protein (23.8%), and total fiber (26.0%).

**Table 4:** Proximate composition of *P. albidus* mycelium cultivated by submerged fermentation compared to basidiome of the same species

Nutritional Component	Mycelium		Basidiome
Lipids	3.4 ± 0.6	2.6 ± 0.8	2.3 ± 0.1
Total Fiber	26.0 ± 0.5	18.5 ± 1.4	44.1 ± 2.3
Protein	23.8 ± 0.1	20.4 ± 1.4	*14.4 ± 0.1
Ash	7.7 ± 0.4	8.1 ± 0.1	7.7 ± 0.1
Carbohydrates	39.0 ± 0.5	50.4 ± 1.0	31.5 ± 4.6
Energy (kcal/100 g)	287 ± 2	381 ± 2	204 ± 10
Reference	[Present Study]	[34]	[35]

Values are presented as mean ± standard deviation of analytical replicates (*n* = 3) obtained from pooled biomass produced in multiple flasks within a single cultivation experiment. *Protein value originally calculated with a factor of 6.25 was recalculated using 4.38. Carbohydrate and energy values were also adjusted accordingly. All values are expressed on a dry basis

The literature offers limited data on the proximate composition of *P. albidus* mycelium produced by submerged fermentation. The study of Kirsch et al. [34], reported the composition of *P. albidus* mycelium cultivated under similar submerged conditions, but using approximately one-sixth of the yeast extract concentration used in the present study. Despite this difference in nitrogen supplementation, the results for protein (20.4%), ash (8.1%), and lipids (2.6%) were remarkably similar to those observed in our study (23.8%, 7.7%, and 3.4%, respectively), suggesting a comparable biochemical profile. These results indicate that *P. albidus* may maintain stable macronutrient composition across a range of cultivation conditions, although further research is needed to explore the influence of medium formulation, strain variability, and process parameters on its nutritional outcomes.

The results obtained were also compared with those of the basidiome produced by the same fungal strain, sourced from the same fungal culture collection [35]. Although mycelium and basidiome differ structurally and in their methods of production, this comparison provides valuable insights into the nutritional potential of mycelial biomass relative to the conventional mushroom.

It is important to emphasize that the cultivation systems are fundamentally different: while the basidiome was obtained on a lignocellulosic solid substrate under controlled environmental temperature and humidity for 45 days, the mycelium was produced by submerged fermentation with controlled temperature and agitation in only 12 days.

When comparing the data, the mycelium showed a significantly higher protein content (23.8%) than the basidiome (14.4%). This result may be attributed to the use of yeast extract as a readily assimilable nitrogen source, as well as to the lower structural complexity of the mycelium compared to the fruiting body tissue. On the other hand, the dietary fiber content was higher in the basidiome (44%) than in the mycelium (26.0%), which reflects the greater presence of organized structures, such as cell walls and thickened tissues specialized in fruiting bodies.

The lower dietary fiber content observed in submerged-cultivated mycelium may therefore be primarily related to differences in developmental stage and tissue organization [36]. In addition, cultivation conditions, including nitrogen availability and medium composition, are known to influence fungal cell wall polysaccharides such as β-glucans [37]. Thus, the nitrogen-rich submerged medium may have favored rapid biomass formation and protein accumulation rather than the extensive deposition of structural polysaccharides. However, since β-glucans were not specifically quantified in this study, this interpretation should be considered a possible explanation rather than a direct mechanistic conclusion.

The total carbohydrate content was higher in the mycelium (39%) compared to the basidiome (31.5%), while lipid and ash contents showed similar values between the two forms.

These results indicate that, although the composition of *P. albidus* mycelium and basidiome reflects their structural and physiological differences, submerged cultivation can be a viable alternative for obtaining biomass with high protein content in a shorter production time. This approach appears promising for food applications, especially in the development of alternative mycelium-based ingredients with nutritional value comparable to that of conventional mushrooms.

From an industrial perspective, commercial and emerging mycoprotein platforms are represented by different fungal species with distinct nutritional characteristics. Reported protein contents are approximately 45% for the ascomycetes *Fusarium venenatum* [8] and *Neurospora crassa* [1], whereas a protein content of approximately 32% has been reported for the basidiomycete *P. pulmonarius* cultivated under submerged conditions [38]. Under the cultivation conditions evaluated in the present study, *P. albidus* mycelium contained 23.8% protein, indicating that although its protein content is lower than that reported for current commercial and emerging mycoproteins, it is within the range observed for edible basidiomycetes cultivated by submerged fermentation. These comparisons also suggest that further optimization of strain selection and cultivation conditions may contribute to increasing the protein content of *P. albidus*, while maintaining its favorable amino acid profile and rapid biomass production.

### Amino Acid Profile, Nutritional and Sensory Potential of *P. albidus* Biomass

#### Composition and Comparison with Other Sources

Table 5 presents the amino acid profile of *P. albidus* mycelial biomass compared to other species of the genus, as well as traditional protein sources such as raw soybean and beef. Although it exhibited considerable levels of leucine (66.8 mg/g), lysine (54.2 mg/g), and threonine (52.5 mg/g), the sum of essential amino acid (∑EAA) (357.1 mg/g) was lower than observed in *P. sapidus* (382 mg/g) [12] and *P. pulmonarius* (416 mg/g) [38].

The latter stood out for its higher leucine (83.4 mg/g) and lysine (88.7 mg/g) contents, exceeding even the values obtained for *P. albidus*. On the other hand, *P. albidus* presented concentrations of threonine, valine, and isoleucine similar to those found in beef and higher than those reported for *P. sapidus*, suggesting that this species may contribute significantly to the nutritional quality of mycelial protein.

Regarding non-essential amino acids (NEAA), *P. albidus* mycelium showed notable levels of glutamic acid (145.0 mg/g) and aspartic acid (102.1 mg/g), totaling 550 mg/g of NEAA. This value was lower than that observed in *P. sapidus* (563 mg/g) and *P. pulmonarius* (584 mg/g), indicating that, despite contributing significantly to flavor and functionality (particularly due to glutamate), *P. albidus* presents a lower overall content. In total, the sum of amino acid (∑AA) (907 mg/g protein) was also slightly lower than that of *P. sapidus* (946 mg/g) and *P. pulmonarius* (1000 mg/g), but still accounted for 90.7% of the protein determined by *N*×4.38. These results reinforce the potential of *P. albidus* as an alternative protein source, although with variations in amino acid composition when compared to related species and traditional protein sources such as soybean and beef. To facilitate the nutritional interpretation of the amino acid composition, the corresponding chemical scores based on the FAO/WHO reference pattern are presented separately in Table 6.

**Table 5:** Essential (EAA) and non-essential (NEAA) amino acid content (mg/g_protein_) of *P. albidus* mycelium compared with other protein sources

Amino Acid	*P*. *albidus*	*P*. *sapidus*	*P*. *pulmonarius*	Raw soybean	Beef
Threonine	52.5	38.2	49.7	39.7	53.3
Valine	49.6	43.3	54.4	44.3	55.5
Methionine	12.6	14.2	8.7	21.2	41.7
Isoleucine	45.0	35.8	46.7	43.8	52.8
Leucine	66.8	64.2	83.4	71.2	98.3
Phenylalanine	37.4	39.0	48.8	80.8	87.6
Lysine	54.2	43.7	88.7	59.6	110
Tryptophan	6.3	7.5	7.7	12.1	12.4
Histidine	32.8	96.5	28.1	26.8	43.1
**∑EAA**	357	382	416	400	555
Aspartic acid	102.1	81.5	101.1		
Glutamic acid	145.0	171.3	137.2		
Serine	50.4	45.7	48.0		
Glycine	58.8	42.5	55.2		
Cystine	6.3	10.6	36.5		
Alanine	68.1	54.7	68.8		
Proline	42.0	40.6	46.7		
Tyrosine	22.7	25.2	34.0		
Arginine	54.6	91.3	58		
**∑NEAA**	550	563	584		
**∑AA**	907	946	1000		
**Reference**	[Present Study]	[12]	[38]	[11]	[11]

∑EAA: sum of essential amino acids; ∑NEAA: sum of non-essential amino acids; ∑AA: the sum of total amino acids. Values for P. albidus were obtained from pooled biomass produced in multiple flasks within a single cultivation experiment and analyzed in a single analytical determination

#### Chemical Score and Limiting Amino Acid

The protein quality of *P. albidus* mycelium was evaluated using the amino acid chemical score, as shown in Table 6. The score was calculated by dividing the value of each essential amino acid by the reference value recommended for adults according to the FAO/WHO standard [18].

A protein is considered of high nutritional value when all EAA have a chemical score equal to or greater than 1.0. If any amino acid presents a value below this threshold, it is considered the limiting amino acid, indicating that the protein may not provide adequate amounts of that nutrient to meet human metabolic requirements [39]. However, it is important to note that the chemical score does not account for protein digestibility. Therefore, the values obtained in this study should be interpreted as preliminary nutritional indicators, since digestibility-corrected methods such as the Protein Digestibility-Corrected Amino Acid Score (PDCAAS) and the Digestible Indispensable Amino Acid Score (DIAAS) were not evaluated.

**Table 6:** Comparison of the chemical scores of essential amino acids in *P. albidus* mycelium, soybean, and beef, highlighting limiting and surplus amino acids

	Chemical Score						
	*P*. *albidus*	*P*. *sapidu*s	*P*. *pulmonarius*	Raw soybean	Beef	Reference Value (mg_AA_/g_protein_)*	
Threonine	2.3	1.7	2.2	1.7	2.3	23	
Valine	1.3	1.1	1.4	1.1	1.4	39	
Methionine + cystine	0.6	0.6	0.4	1.0	1.9	22	
Isoleucine	1.5	1.2	1.6	1.5	1.8	30	
Leucine	1.1	1.1	1.4	1.2	1.7	59	
Phenylalanine + tyrosine	1.0	1.0	1.3	2.1	2.3	38	
Lysine	1.2	1.0	2.0	1.3	2.4	45	
Tryptophan	1.1	1.3	1.3	2.0	2.1	6	
Histidine	2.2	6.4	1.9	1.8	2.9	15	

Chemical scores were calculated by Eq. 5 and the essential amino acid values shown in Table 5. *According to the FAO/WHO [18]

The results show that, although *P. albidus* mycelium contains all essential amino acids, their concentrations relative to the FAO/WHO reference values vary considerably. Threonine exhibited the highest chemical score in *P. albidus* (2.3), following a similar pattern in *P. sapidus* (1.7) and *P. pulmonarius* (2.2). The limiting amino acid was methionine + cystine in all species, with a score of 0.6 in *P. albidus* and *P. sapidus*, and 0.4 in *P. pulmonarius*, indicating a consistent relative deficiency compared to human requirements. Other essential amino acids, such as lysine (1.2, 1.0, and 2.0 for *P. albidus*, *P. sapidus*, and *P. pulmonarius*, respectively) and tryptophan (1.1, 1.3, and 1.3), showed values above 1, suggesting favorable relative levels.

The limitation of methionine + cystine may be associated with sulfur assimilation during fungal metabolism, since these amino acids depend directly on sulfur availability and incorporation pathways. Although sulfate salts were present in the culture medium, sulfur assimilation in fungi requires multiple metabolic conversion steps before incorporation into sulfur-containing amino acids [40]. Therefore, optimization of sulfur supplementation strategies may represent a potential approach to improve amino acid balance and enhance the nutritional quality of the biomass, although this hypothesis still requires experimental confirmation.

#### Sensory Potential Inferred from the Total Amino Acid Profile of *P. albidus* Mycelium

Beyond its nutritional value, AA are also related to sensory potential, as certain free AA are known to be direct precursors of characteristic taste notes such as umami, sweet, or bitter [41]. The sensory inferences presented here are based on the assumption that a portion of the total AA identified may be present in free form, as reported in the literature for other species of the *Pleurotus* genus [5].

Among the amino acids analyzed, glutamic acid (145 mg/g protein) and aspartic acid (102.1 mg/g protein) stood out, totaling 247 mg/g combined, which are precursors of umami taste when present in free form. In addition to these, the mycelium also presented substantial levels of sweet-tasting amino acids, such as alanine (68.1 mg/g), glycine (58.8 mg/g), serine (54.4 mg/g), and proline (42 mg/g). These compounds are recognized for modulating a mild and sweet flavor in mushrooms, which may help balance or mask the perception of potentially bitter amino acids, such as arginine (54.6 mg/g) and tyrosine (22.7 mg/g). The levels of threonine (52.5 mg/g), isoleucine (45 mg/g), and lysine (54.2 mg/g) are also noteworthy, as these AA may contribute to the development of complex flavors through Maillard reactions, particularly under thermal processes [42].

It is important to note that the data presented here refer to the total AA content, comprising both free and protein-bound forms. While only free AA contribute directly to taste perception, protein-bound AA may participate in flavor formation during cooking via hydrolysis or Maillard-type reactions [43]. Therefore, the total AA profile provides a preliminary indication of the sensory and flavor-development potential of *P. albidus* mycelium. Future studies quantifying the free AA fraction will be essential to refine this sensory assessment and better characterize the umami-related contribution.

### Limitations and Future Perspectives

Although the present study demonstrates the potential of *P. albidus* biomass as an alternative protein source, several aspects deserve further investigation before broader industrial and food applications can be fully established. The experiments were performed using a single strain of *P. albidus* under shake-flask cultivation conditions. Therefore, future studies should evaluate strain-dependent variability and investigate cultivation under bioreactor conditions, where oxygen transfer, mixing efficiency and pellet morphology may substantially influence biomass production. In addition, although yeast extract proved to be an effective nitrogen source, the economic feasibility of its use at industrial scale should be further assessed, including the evaluation of lower-cost alternative nitrogen sources.

From a nutritional perspective, the present study focused on protein content and amino acid composition. However, additional quality indicators, including protein digestibility (e.g., in vitro digestibility, PDCAAS or DIAAS), as well as fungal bioactive components such as β-glucans, chitin, ergosterol, vitamins and minerals, were beyond the scope of this work and should be investigated in future studies. Furthermore, sulfur-containing amino acids were identified as the limiting amino acids according to the FAO/WHO reference pattern, suggesting that formulation strategies combining *P. albidus* biomass with complementary protein sources may further improve the nutritional quality of food products.

Finally, although the compositional characteristics indicate promising potential for food applications, technological and sensory properties, consumer acceptance and comparisons with commercially available mycoproteins remain important topics for future investigation before direct industrial implementation can be fully established.

## Conclusions

This study demonstrated that *P. albidus* mycelium can be efficiently produced under submerged conditions, particularly using yeast extract as a nitrogen source, yielding biomass with 23.8% protein. All essential amino acids were detected, although sulfur-containing amino acids were limiting. The total amino acid profile also suggests potential for future sensory exploration. These attributes support its further investigation as a sustainable and functional protein ingredient for food applications. Beyond its nutritional and technological properties, *P. albidus* represents a promising yet underexplored fungal species. The results highlight the value of investigating lesser-known strains to diversify the sources of alternative proteins and drive innovation in the development of novel, clean-label food products.

## Data Availability

The datasets generated and/or analyzed during the current study are available from the corresponding author upon reasonable request.

## Declarations

### Artificial Intelligence

The authors used a generative artificial intelligence tool solely to assist with language refinement, improvement of text clarity, and editorial revision during manuscript preparation. The scientific content, experimental design, data analysis, interpretation of results, and final conclusions were developed and critically reviewed by the authors, who take full responsibility for the content of this manuscript.
